# Cost-effectiveness analysis of vaccination strategies against meningococcal disease for children under nine years of age in China

**DOI:** 10.1080/21645515.2024.2313872

**Published:** 2024-02-13

**Authors:** Haonan Zhang, Haijun Zhang, Hai Fang

**Affiliations:** a School of Public Health, Peking University, Beijing, China; b China Center for Health Development Studies, Peking University, Beijing, China; c Department of International Health, Johns Hopkins Bloomberg School of Public Health, Baltimore, MD, USA; d Peking University Health Science Center-Chinese Center for Disease Control and Prevention Joint Center for Vaccine Economics, Peking University, Beijing, China; e Institute for Global Health and Development, Peking University, Beijing, China

**Keywords:** Meningococcal disease, vaccination strategies, meningococcal vaccines, cost-effectiveness analysis, economic evaluation, China

## Abstract

Meningococcal vaccination strategies in China are intricate, including multiple vaccines targeting different serogroups. The current National Immunization Program (NIP) includes two polysaccharide vaccines for serogroups A and C (MPV-A and MPV-AC), covering limited serogroups and requiring adaptation. This study aims to evaluate the cost-effectiveness of replacing the current strategy with alternative strategies utilizing non-NIP vaccines to inform policy decisions. From a societal perspective, a decision tree-Markov model was constructed to simulate the economic and health consequences of meningococcal disease in a 2019 birth cohort with four vaccination strategies. Epidemiology, vaccine efficacy, cost, and other parameters were derived from previous studies. We conducted sensitivity analyses to assess the robustness of the findings and explored prices for non-NIP vaccines that enable cost-effective strategies. Compared to the current strategy, alternative strategies using quadrivalent polysaccharide vaccine (MPV-4), bivalent conjugate vaccine (MCV-AC), and quadrivalent conjugate vaccine (MCV-4) could avoid 91, 286, and 455 more meningococcal cases. The ICERs were estimated at approximately $250 thousand/QALY, $450 thousand/QALY, and $1.5 million/QALY, all exceeding the threshold of three times GDP per capita. The alternative strategies were not cost-effective. However, if vaccine prices were reduced to $3.9 for MPV-4, $9.9 for MCV-AC, and $12 for MCV-4, the corresponding strategy would be cost-effective. The current meningococcal vaccination strategy in China could effectively prevent the disease at a low cost, but with limited serogroup coverage. Strategies using MPV-4, MCV-AC, or MCV-4 could increase health benefits at a substantial cost, and might become cost-effective if vaccine prices decrease.

## Introduction

Meningococcal disease, caused by the obligate human pathogen Neisseria meningitidis (also referred to as meningococcus), poses a significant threat to the wellbeing of humans, especially that of children. The Gram-negative aerobic diplococcus can be classified into 12 serogroups, with serogroup A (MenA), serogroup B (MenB), serogroup C (MenC), serogroup Y (MenY), serogroup W (MenW) and serogroup X (MenX) being the main serogroups responsible for current cases.^1–3^ The meningococcal disease manifests primarily as meningitis and septicemia (meningococcemia), with an overall mortality rate of 8–14%. Those who survive may suffer from permanent sequelae such as seizures, hearing loss, and limb amputation.^4^ The huge cost of treatment and loss of labor can impose a heavy financial burden on families. Prevention of meningococcal disease can avoid tragedies and promote national health, and vaccines are the most effective measure available. The development of appropriate and effective immunization strategies against meningococcal disease is crucial for countries and regions.

In China, the incidence of meningococcal disease has remained below 1/100,000 since the 1990s, and no pandemics or outbreaks have occurred since 2003.^5^ Currently, the primary serogroups responsible for the disease are MenB, MenC, and MenW.^6^ MenC was the predominant circulating serogroup before 2014, accounting for 59.6% of cases, followed by MenW at 24.4%. After 2015, meningococcal cases caused by MenB exhibited an upward trend, with its share exceeding half. The share of cases due to MenC decreased to 22.8% (95% CI: 18.2%–27.4%), followed by MenW (16.8%) and MenA (4.7%). The incidence due to MenA and MenC has decreased significantly due to the mass vaccination induced since the inclusion of MPV-A in the NIP in 2003 and MPV-AC in 2008.^7^ Of all meningococcal disease cases, the incidence among children aged 0–5 years and 6–10 years in China account for 29.6% and 28.9% respectively, while individuals aged 18 and above constitutes 19.2%.^7^ Children face a notably high risk of contracting Neisseria meningitidis. The NIP offers two doses of polysaccharide vaccine against MenA (MPV-A) at 6 and 9 months of age and two doses of bivalent polysaccharide vaccine against MenA and MenC (MPV-AC) at 3 and 6 years old, free of charge to children. In addition to the two vaccines in the NIP, several other meningococcal vaccines have been approved for use in children, including a bivalent conjugate vaccine against MenA and MenC (MCV-AC), a quadrivalent polysaccharide vaccine against serogroups A, C, Y and W (MPV-4), and a quadrivalent conjugate vaccine against serogroups A, C, Y, and W (MCV-4). These non-NIP vaccines are available to families at their own expense for children of eligible age.

Compared to polysaccharide vaccines, conjugate vaccines exhibit improved immunogenicity outcomes, efficacy, and duration of protection, with a favorable safety profile supported by phase III clinical trials.^5,8^ In a comparative safety and immunogenicity trial of meningococcal vaccines in children aged 2–10 years, all four serogroups in the quadrivalent conjugate vaccine group showed significantly higher rates of functional antibody seroconversion percentages than the quadrivalent polysaccharide vaccine group.^9^ A review of effectiveness studies on meningococcal vaccines showed effectiveness rates of 65% ~ 83.7% for the polysaccharide vaccine and 66% ~ 100% for the conjugate vaccine, and concluded that conjugate vaccine had demonstrated higher effectiveness with longer-lasting protection.^10^ The strengths of conjugate vaccine in terms of immunological memory and protection have also been documented in toddlers who do not respond well to polysaccharide vaccines.^11^ Given the comprehensive and effective protection offered by polyvalent conjugate vaccines, global trends in vaccination strategies against meningococcal disease have shifted toward the replacement of monovalent vaccines with polyvalent vaccines and the replacement of polysaccharide vaccines with conjugate vaccines, and many countries have incorporated MCV-4 into their NIPs, such as the US, the UK, Canada, etc. However, the NIP in China only includes polysaccharide vaccines that provide protection against limited serogroups (MenA and MenC), thereby leaving the risk of potential outbreaks by other serogroups as well as suboptimal protection in younger age groups (i.e., infants and toddlers).

With the aim of improving population health, the adaptation of vaccination strategy in line with global trends should be put on the agenda in China. Such decisions on immunization strategies require supporting evidence in the local context, including the health outcomes of strategies from an epidemiological perspective and the costs of strategies from an economic perspective to determine whether the investment in immunization is worthwhile. In this regard, literature on the economic evaluation of meningococcal vaccination is available in many countries, while the existing studies in China are quite limited, with only three studies examining the current strategy adopted by NIP at the provincial level.^12–15^ No economic evaluations have yet been conducted on vaccination strategies using other non-NIP vaccines on the market. Therefore, this study aimed to conduct a comprehensive economic evaluation of multiple meningococcal vaccination strategies incorporating various vaccines into the NIP at the national level, and to determine whether replacing the current strategy with alternative strategies is desirable in the context of China.

## Methods

The cost-effectiveness analysis was performed to assess the cost and health outcomes of various meningococcal vaccination strategies for children under nine years of age from a societal perspective. The objective was to compare the current strategy with alternative strategies that incorporated non-NIP vaccines into the NIP, and determine the incremental cost, incremental effectiveness, and incremental cost effectiveness ratios (ICERs).

### Vaccination strategies

Four different vaccination strategies were examined, as shown in Table 1. The *Current Practice* was established as the baseline against which each of the other three strategies using alternative vaccines available in private market was compared. The other three strategies assumed that private market vaccines would be integrated into NIP to ensure free vaccination for children. The NIP vaccines could reach a coverage rate of 99% among the targeted population in China.^16^ The alternative strategies using non-NIP vaccines were developed based on the recommended immunization schedule indicated in the vaccine instructions and the common practice for self-funded vaccination. The protection for children provided by the meningococcal polysaccharide vaccine could last up to three years,^17,18^ and the protection provided by the conjugate vaccine can last for about five years.^19–22^ Thus, *Current Practice* could protect children up to nine years of age. The alternative strategies incorporating other privately marketed vaccines should attain the same duration of protection. In the strategies using conjugate vaccines, children were scheduled to receive a dose of MPV-AC at six years of age.

**Table 1.** Vaccination strategies in preventing meningococcal disease.

Strategy	Vaccines in NIP	Vaccination schedule
*Current Practice*	MPV-A MPV-AC	2 doses of MPV-A at 6 months and 9 months of age 2 doses of MPV-AC at 3 years and 6 years of age
*Universal MPV4*	MPV-A MPV-4	2 doses of MPV-A at 6 months and 9 months of age 2 doses of MPV-4 at 3 years and 6 years of age
*Universal MCV-AC*	MCV-AC MPV-AC	2 doses of MCV-AC at 6 months and 7 months of age 1 dose of MPV-AC at 6 years of age
*Universal MCV-4*	MCV-4 MPV-AC	3 doses of MCV-4 at 3 months, 4 months, and 5 months of age 1 dose of MPV-AC at 6 years of age

The current practice was adopted as the benchmark strategy and was compared to each of the other three alternative strategies using non-NIP vaccines.

### Model

A decision tree-Markov model was developed in TreeAge Pro 2019 on a cohort of 14.65 million Chinese newborns in 2019. Simulations were conducted on a yearly cycle, from birth to the age of 9 years. The study adopted a life expectancy of 77.3 years for the 2019 birth cohort as the time horizon, and took into account lifetime costs and impacts. Herd immunity was not included in this study, given the current high coverage of NIP vaccines and the relatively low incidence of meningococcal disease in China. With the vaccine coverage of NIP, almost all children would get vaccinated, and this leads to only a low number of children in the cohort who need indirect protection by herd immunity. The impact of serogroup replacement on the incidence of meningococcal disease caused by different serogroups were also not included due to the limited duration of the simulation. All costs and health outcomes were discounted to 2019 at a discount rate of 3%, according to the WHO guidelines.^23^

**Figure 1.**
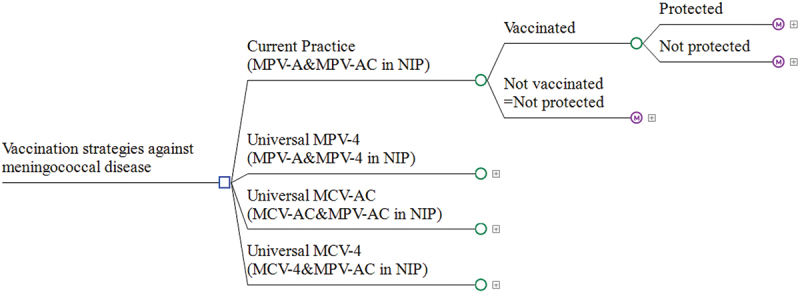
Decision tree-markov model.

**Figure 2.**
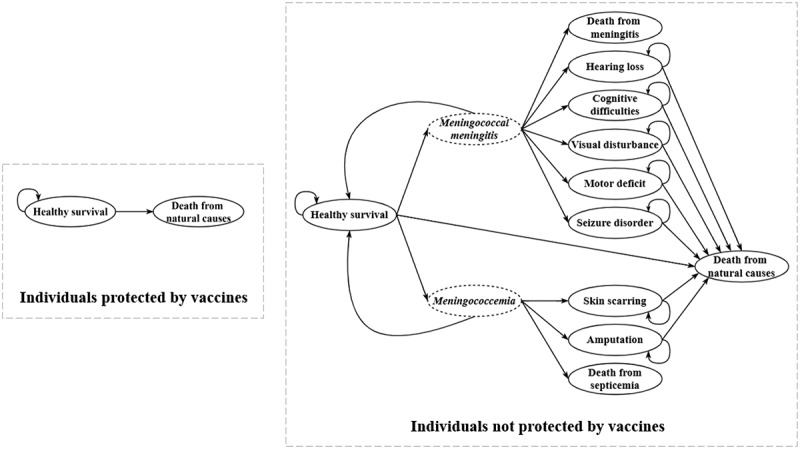
Markov chains simulating the health status of individuals.

The structure of the model was illustrated in Figure 1. The subtree of the decision tree was identical for each strategy, and the effect of vaccine protection varies across strategies. Markov chains were established separately for the protected and the unprotected, and the states in Markov chain were partitioned according to the health status of the population (Figure 2). The model comprised eleven states for the unprotected population, with “meningococcal meningitis” and “meningococcemia” serving as intermediate stages added to facilitate understanding of the transition process. The unprotected individuals were initially in a healthy survival state and at risk of meningococcal disease. Given the severity of meningococcal disease and the availability of healthcare services in China, all cases were assumed to receive treatment in hospital, and could recover to the healthy survival state, or survive with sequelae, or die from the disease.

Individuals protected by vaccines refer to those who were vaccinated and immunized, and individuals not protected by vaccines include those who have not been vaccinated and those for whom vaccination has not produced a protective effect.

### Parameters input

The data sources for input parameters are summarized in Table 2. The incidence of meningococcal meningitis was extracted from the Global Burden of Disease (GBD) database for China in 2019. Due to difficulties in distinguishing meningococcemia from septicemia of other etiologies in clinical practice, data on the incidence of meningococcemia were scarce globally and in China, potentially leading to an underestimation of the disease burden. This study used data from a geographically similar Asian country to estimate the incidence of meningococcemia in China.^24^ The proportion of cases attributed to different serogroups in the overall cases was derived from a study by the China CDC.^25^ The probabilities of sequelae and case fatality rates for meningococcal meningitis and meningococcemia were obtained from systematic reviews, while the mortality rates for natural causes in 2019 was collected from the Chinese Health Statistics Yearbook.^26–29^

**Table 2.** Parameter inputs in the model.

Variable	Base case	Range	Distribution	Reference
EPIDEMIOLOGY				
Incidence of meningococcal meningitis per 100,000	1.9028	0.8762–3.7565	Beta	GBD
Incidence of meningococcemia per 100,000	0.8034	0.3700–1.5861	Beta	GBD Horino et al.^24^
*Serogroup distribution of meningococcal cases (%)*				
Proportion of MenA in all cases	4.73	Base ± 25%	Beta	Li et al.^25^
Proportion of MenC in all cases	22.97	Base ± 25%	Beta	Li et al.^25^
Proportion of MenY in all cases	1.69	Base ± 25%	Beta	Li et al.^25^
Proportion of MenW in all cases	6.08	Base ± 25%	Beta	Li et al.^25^
*Sequelae in meningitis (%)*				
Hearing loss	4.6	3–7.3	Beta	Edmond et al.^26^
Cognitive difficulties	2.9	1–7.2	Beta	Edmond et al.^26^
Visual disturbance	2.7	1.1–4.1	Beta	Edmond et al.^26^
Motor deficit	1.8	1–4.1	Beta	Edmond et al.^26^
Seizure disorder	0.9	0.1–2	Beta	Edmond et al.^26^
*Sequelae in Meningococcemia (%)*				
Skin scarring	9	Base ± 25%	Beta	Vyse et al.^27^
Amputations	28.6	Base ± 25%	Beta	Horino et al.^24^ Martinón-Torres et al.^28^
Case fatality rate, meningococcal meningitis (%)	4.8	2.86–7.31	Beta	Wahl et al.^29^
Case fatality rate, meningococcemia (%)	26.3	13.2–40	Beta	Horino et al.^24^
*All-cause mortality rate (‰)*				
<1y	2.8	Base ± 25%	Beta	Health Statistical Yearbook
1–4y	1.2	Base ± 25%	Beta	Health Statistical Yearbook
5–9y	0.14	Base ± 25%	Beta	Population census
COST (RMB)				
*Cost of vaccination program*				
MPV-A price, per dose	0.209	0.157–0.261	Gamma	China CDC
MPV-AC price, per dose	1.728	1.296–2.160	Gamma	China CDC
MPV-4 price, per dose	16.815	6.958–21.019	Gamma	Centralized procurement
MCV-AC price, per dose	22.904	10.872–28.629	Gamma	Centralized procurement
MCV-4 price, per dose	60.883	12.177–102.486	Gamma	Centralized procurement
Syringe price	0.174	0.036–0.652	Gamma	Centralized procurement
Vaccine service cost, per dose	3.714	Base ± 25%	Gamma	Yu et al.^30^
Indirect cost, per dose	6.303	Base ± 25%	Gamma	Yu et al.^30^
Vaccine wastage (%)	5	Base ± 25%	Beta	WHO^31^
*Economic burden of the disease*				
Cost of Meningococcal meningitis	8373.310	Base ± 25%	Gamma	Liu et al.^32^
Cost of Meningococcemia	11866.435	Base ± 25%	Gamma	Martinón-Torres et al.^28^ Liu et al.^32^
Cost of cognitive difficulties	36544.495	Base ± 25%	Gamma	CHIRA
Cost of seizure disorder	832.911	Base ± 25%	Gamma	CHIRA
Cost of hearing loss	22624.275	Base ± 25%	Gamma	CHIRA
Cost of motor deficit	1755.341	Base ± 25%	Gamma	CHIRA
Cost of visual disturbance	6435.342	Base ± 25%	Gamma	Yang et al.^34^
Cost of amputation	4659.918	Base ± 25%	Gamma	Wang et al.^33^
Cost of productivity lost due to death	111626.703	Base ± 25%	Gamma	Population census
Discount rate (%)	3	2.25–5	Uniform	WHO^23^
USD to RMB exchange rate in 2019	6.8985			National Bureau of Statistics
VACCINE EFFICACY (%)				
MPV-A vaccine efficacy-A	55.56	Base ± 25%	Beta	Xie et al.^8^
MPV-AC vaccine efficacy-A	75.36	Base ± 25%	Beta	Tang et al.^35^
MPV-AC vaccine efficacy-C	94.2	Base ± 25%	Beta	Tang et al.^35^
MPV-4 vaccine efficacy-A	82.15	Base ±25%	Beta	Xie et al.^8^
MPV-4 vaccine efficacy-C	90.57	Base ± 25%	Beta	Xie et al.^8^
MPV-4 vaccine efficacy-Y	50.84	Base ± 25%	Beta	Xie et al.^8^
MPV-4 vaccine efficacy-W	54.88	Base ± 25%	Beta	Xie et al.^8^
MCV-AC vaccine efficacy-A	87.43	Base ± 25%	Beta	Xie et al.^8^
MCV-AC vaccine efficacy-C	92.81	Base ± 25%	Beta	Xie et al.^8^
MCV-4 vaccine efficacy-A	91.42	Base ± 25%	Beta	Xie et al.^8^
MCV-4 vaccine efficacy-C	88.76	Base ± 25%	Beta	Xie et al.^8^
MCV-4 vaccine efficacy-Y	88.17	Base ± 25%	Beta	Xie et al.^8^
MCV-4 vaccine efficacy-W	99.41	Base ± 25%	Beta	Xie et al.^8^
UTILITY				
Utility of Health	1	–	N/A	
Utility of Death	0	–	N/A	
Utility of meningitis	0.9768	0.597–1	Beta	Bennett et al.^36^
Utility of meningococcemia	0.9921	0.7825–1	Beta	Bennett et al.^36^
*Utility of Sequelae*				
Cognitive difficulties	0.62	0.51–0.73	Beta	Oostenbrink et al.^37^
Seizure disorder	0.83	0.75–0.91	Beta	Oostenbrink et al.^37^
Hearing loss	0.91	0.83–1	Beta	Oostenbrink et al.^37^
Motor deficit	0.67	0.55–0.79	Beta	Oostenbrink et al.^37^
Visual disturbance	0.26	0.19–0.33	Beta	Brown et al.^38^
Skin scarring	1	Base ±25%	Beta	Blakeney et al.^39^
Amputation	0.69	0.69–0.8	Beta	Erickson et al.^40^
THRESHOLD				
China’s GDP per capital in 2019 (RMB)	70078			National Bureau of Statistics

The cost analysis considered both the cost of the vaccination program and the economic burden of the disease. The cost of vaccination comprised vaccine procurement, syringe procurement, vaccine services, and indirect costs. Prices for NIP vaccines followed the recommendations provided by the China CDC, at $0.209 per dose for MPV-A and $1.728 per dose for MPV-AC. Prices for MCV-AC ($22.904 per dose), MPV-4 ($16.815 per dose), and MCV-4 ($60.883 per dose) and the price of syringe followed data from centralized procurement in China. The cost of vaccine services, including transportation, supervision, staff training, personnel wage, and cold chain, was derived from a survey study conducted by the China CDC.^30^ The same study also estimated the indirect costs associated with vaccination, including the productivity loss of parents accompanying children and transportation expenses. The wastage rate for vaccines was assumed to be 5% in accordance with WHO guidelines.^31^

The economic burden of disease in this study included the cost of treating meningitis or meningococcemia, costs arising from sequelae, and loss of productivity due to sequelae or death. China CDC have investigated the economic burden of three bacterial meningitis cases, from which the cost for meningococcal meningitis was obtained.^32^ Due to the lack of definitive clinical diagnosis and cost data for meningococcemia, the cost for meningococcemia was estimated from the cost for meningitis in China, with reference to a review providing the cost for meningitis and meningococcemia in the U.S.^28^ Costs for the sequelae of meningococcal disease were derived from the China Health Insurance Research Association (CHIRA) and relevant studies conducted in China.^33,34^ Costs for cognitive difficulties covered treatment and special education expenses, costs for hearing loss covered cochlear implants and maintenance, and costs for visual impairment was calculated using a discounted annual additional cost for individuals with visual impairment. The loss of lifetime productivity due to disability and death was estimated using data from the 6th Population Census in China.

Limited evidence has been found regarding the efficacy of meningococcal vaccines, despite their availability for years. According to the ACIP report on the prevention and control of meningococcal disease, the low incidence of meningococcal disease has precluded the assessment of clinical efficacy, and immunogenicity data could serve as a proxy for efficacy. In China, various manufacturers may produce the same type of vaccine, with varying efficacies and vaccination schedules. In such cases, the vaccination schedule and the immunogenicity data of the vaccine with the highest market share were adopted as a representative.^8,35^

The utility value of 1 was assigned to represent health, while a value of 0 was assigned to death. The utilities for meningitis, meningococcemia and their sequelae were obtained from the existing literature.^36–40^ In particular, the utility value for meningococcemia was estimated using the utility for septicemia in general and may therefore be subject to overestimation.

### Sensitivity analyses

The one-way deterministic sensitivity analyses were conducted to capture the uncertainties in input variables and verify the robustness of the results. The range of values for the parameters were summarized in Table 2, and a deviation of ± 25% was applied to parameters without available references on ranges. The effects of variations in specific parameter on the results were depicted using tornado diagrams. A probabilistic sensitivity analysis (PSA) was conducted by assigning distributions to key parameters and performing Monte Carlo simulations with 1000 iterations. The results were presented in the form of the cost-effectiveness acceptability curve. Moreover, the study explored the precise prices of non-NIP vaccines that would make the alternative vaccination strategy cost-effective.

## Result

### Health impact

The health impacts and costs of various vaccination strategies are presented in Table 3. Our analysis indicated that, for the whole birth cohort from birth to nine years of age, 3066 cases of meningococcal disease, 385 sequelae and 340 deaths would arise under the current strategy. In comparison, alternative strategies demonstrated the potential to reduce the number of sequelae and deaths to varying degrees. The *Universal MPV-4* strategy reduced the number of cases by 91, sequelae by 11 and deaths by 10 compared to the current strategy. The *Universal MCV-AC* strategy yielded a reduction of 286 cases, 36 sequelae cases and 31 deaths. The *Universal MCV-4* strategy was the most effective one, with a reduction of 455 cases, 57 sequelae and 50 deaths. Although there was no significant difference in discounted quality-adjusted life years (QALYs) among individuals, for the whole cohort, replacing *Current Practice (MPV-AC)* with *Universal MPV-4*, *Universal MCV-AC*, or *Universal MCV-4* could result in an additional 366.25, 1128.05, and 1801.95 QALYs, respectively. Replacement of the current meningococcal vaccination strategy with the strategy incorporating MCV-4 and MPV-AC has the potential to deliver the greatest health benefits.

**Table 3.** Incremental cost and effectiveness of strategies compare to *Current practice*.

	Current Practice	Universal MPV-4	Universal MCV-AC	Universal MCV-4
Meningococcal disease (cases)	3066	2975 (⇩91)	2780 (⇩286)	2611 (⇩455)
Sequelae (cases)	385	374 (⇩11)	349 (⇩36)	328 (⇩57)
Death (cases)	340	330 (⇩10)	309 (⇩31)	290 (⇩50)
Individual effectiveness (QALYs)	29.964177	29.964202 (⇩0.000025)	29.964254 (⇧0.000077)	29.964299 (⇩0.000122)
Incremental effectiveness (QALYs)	–	366.25	1128.05	1801.95
Individual Cost (USD)	744.98	751.17 (⇩6.19)	779.95 (⇧34.97)	932.39 (⇩187.41)
Incremental cost (USD)	–	90,701,898.74	512,362,624.2	2,745,531,291
ICER (USD/QALY)	–	251,085.50	452,746.56	1,527,100.66

### Costs

The *Current Practice* had the lowest cost, averaging at $740 per individual. The implementation of the alternative strategies resulted in an increase in cost. The replacement of *Current Practice* with *Universal MPV-4* would increase the cost by $6.2 per child and $91 million for the cohort, and the replacement with *Universal MCV-AC* would increase the cost by $35 per child and $510 million for the cohort. The highest cost would result from the implementation of *Universal MCV-4*, with an increase of $180 per child and $2.7 billion for the cohort (Table 3).

### Cost-effectiveness analysis

If *Universal MPV-4* was adopted to replace the *Current Practice*, incorporating MPV-A and MPV-4 into the NIP to prevent meningococcal disease, the ICER would be $250 thousand per QALY gained. The ICERs for *Universal MCV-AC* and *Universal MCV-4* were $450 thousand/QALY and $1.5 million/QALY respectively (Table 3). A threshold of three times the GDP per capita in China in 2019 ($30,475) was applied in the analysis, and none of the strategies were deemed cost-effective as the calculated ICERs exceeded the threshold.

### Sensitivity analyses

**Figure 3.**
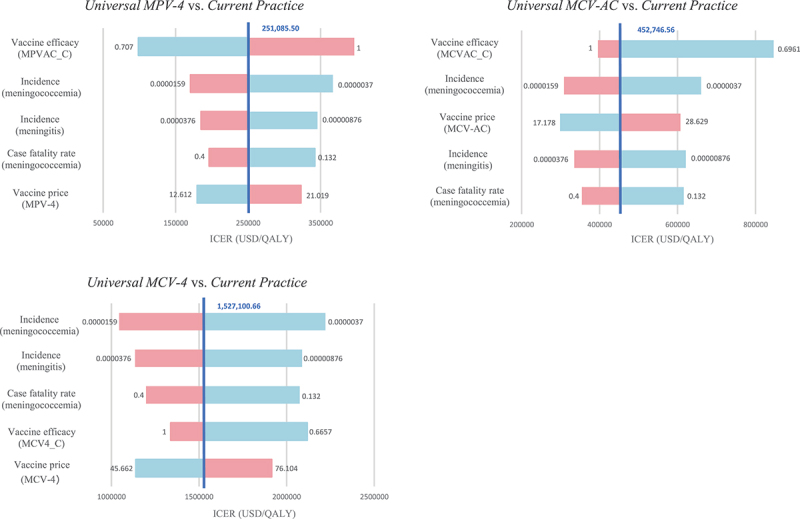
Tornado diagrams for replacing *Current practice* with *universal MPV-4*, *universal MCV-AC*, and *universal MCV-4*.

**Figure 4.**
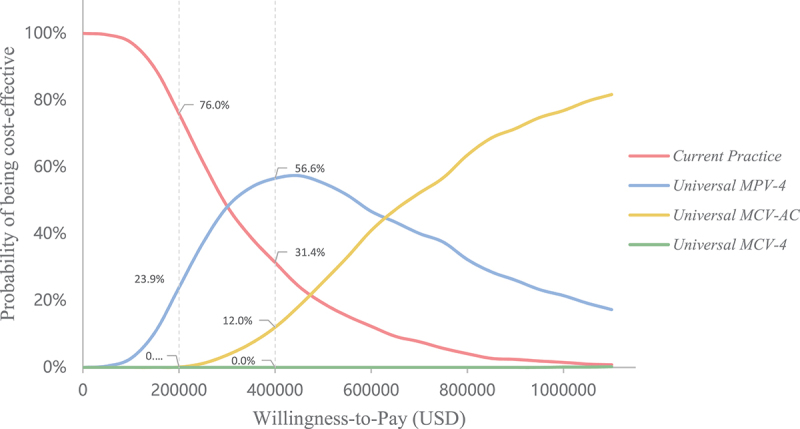
Cost-effectiveness acceptability curves for *Current practice, universal MPV-4*, *universal MCV-AC*, and *universal MCV-4*..

The primary factors influencing the outcomes were found to be the same across all three alternative strategies, including the price of non-NIP vaccines, vaccine efficacy, incidence of meningococcal meningitis, incidence of meningococcemia, and case fatality rate of meningococcemia, although their impact differed (Figure 3). Despite variations in the key parameters over the range, the results of the three alternative strategies not being cost-effective compared to the current strategy remained unchanged. The results of the PSA revealed that the current strategy had the highest probability of being cost-effective within a certain range of thresholds (Figure 4). At a threshold of $200 thousand/QALY, the estimated probability of cost-effectiveness was 76.0% for the current strategy, 23.9% for the *Universal MPV-4*, and 0.1% for the *Universal MCV-AC*. At a threshold of $400 thousand/QALY, the probability of cost-effectiveness was higher for the *Universal MPV-4* than for the current strategy, while the probability remained at 0 for the *Universal MCV-4*.

The price of vaccines was identified as a key factor affecting the ICERs, and was the only modifiable parameter among the primary influencing factors. It was noteworthy that vaccines would often experience price discounts when they were incorporated into the NIP. In light of this, we explored the ICERs for alternative strategies at different prices of non-NIP vaccines and summarized in supplementary material (Figure S1). To make the alternative strategies cost-effective, the price of the MPV-4 should be reduced to $3.9 (76.8% reduction), the price of MCV-AC to $9.9 (56.8% reduction), and the price of MCV-4 to $12 (80.3% reduction).

## Discussion

This study is the first to evaluate the cost-effectiveness of replacing the current NIP strategy with alternative meningococcal vaccination strategies using non-NIP vaccines at a national level in China. The results indicated that for the immunization program targeted children aged 0–9 years in China, the vaccination strategy using 3 doses of MCV-4 and 1 dose of MPV-AC delivered the highest health benefits, followed by a strategy utilizing 2 doses of MCV-AC and 1 dose of MPV-AC, and then a strategy with 2 doses of MPV-A and 2 doses of MPV-4, all delivering health improvements over the current strategy with 2 doses of MPV-A and 2 doses of MPV-AC. The cost of these vaccination strategies rose with health benefits, and none of the three alternative strategies using non-NIP vaccines were cost-effective compared to current strategy. Moreover, sensitivity analyses indicated that variations in the parameters did not affect the result that the ICERs for strategy replacements remained above the threshold, demonstrating the robustness of the results. However, under the current circumstances, reduced prices for non-NIP vaccines could make the alternative strategies cost-effective, with the required prices being $3.9 for MPV-4, $9.9 for MCV-AC, and $12 for MCV-4.

Prior studies evaluating the cost-effectiveness of meningococcal vaccines have had limited comparability to the present study. MPV-A, MPV-AC, MPV-4 and MCV-AC are rarely utilized in other countries at present. A cost-benefit analysis of the MPV-4 was conducted among college students in the US in 1995, and concluded that the strategy was not desirable.^41^ Another study in West Africa compared mass vaccination and reactive vaccination with MPV-AC and found that mass vaccination with MPV-AC was more cost-effective.^42^ The diverse timing, populations, strategies, and epidemiological characteristics and levels of development in the areas studied frustrate the comparison of the findings of these studies with those of the present study. Several studies have evaluated the economics of MCV-4, an emerging and effective polyvalent conjugate vaccine. In Canada, three studies have compared the use of MCV-4 to that of MCV-C. Two revealed that replacement with MCV-4 was not cost-effective, which is consistent with the findings of the present study that strategy with MCV-4 yielded the highest cost per QALY gained.^43,44^ The remaining one observed lower ICERs for the MCV-4 strategy in adolescents than in infants, and the strategy of vaccinating adolescents with MCV-4 and vaccinating infants with MCV-C was even less costly than the vaccinating both populations with MCV-C.^45^ This suggests that cost-effective analysis of meningococcal vaccination strategies could be conducted for different populations in the future, and further support the decision-making for expanding the NIP coverage to adolescents for more universal protection against meningococcal disease in China. Additionally, considering the varying economic development and incidence of meningococcal disease in different provinces, meningococcal vaccination strategy could be developed at provincial level. Such decisions could be based on the provincial economic evaluations of meningococcal vaccination strategies to implement appropriate strategies in different areas.

In some countries, strategies utilizing MCV-4 were cost-effective. Studies conducted in the Netherlands and Norway evaluated the inclusion of MCV-4 into NIP vaccination among adolescents and all concluded cost-effectiveness under a high willingness-to-pay threshold.^46,47^ The Norwegian study in 2021 adopted a threshold of €86 thousand/QALY, which was formulated based on the local GDP per capita and higher than the threshold in the present study. This study also assumed a 50% price discount for MCV-4 included in the NIP, reducing the ICERs and contributing to the cost-effectiveness.^47^ Given the bargaining power of national procurement and high demand for NIP vaccines in China, the prices of non-NIP vaccines are likely to decrease after their inclusion in the NIP, leading to lower ICERs and potential cost-effectiveness. Moreover, the establishment of a national willingness-to-pay threshold in China would provide a local benchmark for economic evaluations, incorporating reference to WHO thresholds, the status of national economic development, and the importance attached to population health. A study by Kuznik et al. assessed MCV-4 as an alternative to MCV-A in 26 African countries in the meningitis belt and found that the MCV-4 strategy was cost-effective in 14 countries at an incidence of 50 per 100,000/year, and in all 26 countries at an incidence of 150 per 100,000/year.^48^ With higher incidence of meningococcal disease, more cases could be averted through the use of non-NIP vaccines like MCV-4. Then, incremental effectiveness could increase, while the increased cost of universal vaccination program due to expensive private market vaccines could be offset more by the decreased economic burden caused by the averted cases, allowing for reduced incremental costs with other parameters fixed. Therefore, the low incidence of meningococcal disease in China largely explains the high ICERs observed in this study. Data from the National Notifiable Disease Reporting System (NNDRS) between 2006 and 2014 indicate a recorded incidence rate of 0.047 cases per 100,000 individuals for meningococcal disease, and this was lower than estimated incidence.^6^ The system might suffer from under-ascertainment, and meningococcal cases may occur without presentation to a health facility. Economic evaluation using underestimated incidence would lead to underestimated health and economic benefits of the vaccine, resulting in less perceived cost-effectiveness.

The current preventive vaccination strategy with MPV-A and MPV-AC in the NIP has achieved relatively favorable control and prevention with low cost, maintaining a low incidence of meningococcal disease in China. However, given the devastating costs of the disease to families in treatment and productivity losses, efforts are needed to further reduce the incidence of the disease and this exceeds the capability of the current strategy. The current strategy cannot prevent disease caused by serogroups other than MenA and MenC, leaving a risk of meningococcal infections from other serogroups. The findings of this study indicate that all three strategies using non-NIP vaccines could reduce incidence, with MCV-4 delivering the greatest health benefit gains, followed by MCV-AC and MPV-4. None of the three strategies was deemed cost-effective compared to the current practice, but cost-effectiveness may be achieved if manufacturers offer price discounts for the inclusion of vaccines in the NIP for universal vaccination. When the price of a non-NIP meningococcal vaccine is lower than the price for cost-effectiveness illustrated earlier, then the corresponding vaccine should be considered for inclusion in the NIP to improve population health at a reasonable cost. Furthermore, the predominant prevalent serogroup is MenB, which responsible for up to 52.4% of cases in China.^7^ No vaccine against MenB has yet been approved for marketing in mainland China. Among the 58 countries that have approved MenB vaccines, 15 have included MenB vaccination in their immunization programmes, targeting at least one age group (primarily infants).^49^ The cost-effectiveness analysis in the UK underscored the value of infant MenB vaccination, demonstrating its cost-effectiveness at a threshold of £20,000 per QALY gained. Infant vaccination was found to be the most effective short-term strategy for reducing MenB cases, particularly when herd effects were included.^50^ The introduction of the MenB vaccine into China would potentially result in a significant reduction in the number of meningococcal cases and alleviate the associated economic burden. Prospective economic evaluations could be conducted to assess the health and economic impact of approving MenB vaccine in the private market or further incorporating it into the NIP.

Limitations of this study must be acknowledged. First, the data on meningococcemia in the model may not accurately reflect the true values, as no local data are currently available. Data from a Japanese study was used to estimate the incidence of meningococcemia in China, but the epidemiology of meningococcal disease in Japan might differ from that in China, despite the geographical proximity. The results of sensitivity analyses demonstrated that the incidence of meningococcemia was a significant factor affecting the ICERs, but variations in the parameter did not alter the primary outcome that the strategy replacements are not cost-effective. Second, the waning of vaccine efficacy was not included in this study. It was assumed that the protection for vaccinated children would last until the next vaccination. No studies have yet explored the waning of efficacy for meningococcal vaccines available in China, and limited data could be found on the duration of protection for meningococcal vaccines. Although measurement of vaccine efficacy in the model was simplified due to the lack of reliable data, sensitivity analyses suggested that variations in vaccine efficacy did not affect the findings. Third, the decision tree-Markov model used in the study is a static model. The probability of an individual switching between health states does not vary with the number of infections and over time, and therefore the model may not capture the impact of herd immunity. Unvaccinated populations could be protected from meningococcal disease due to vaccination in others, and the number of cases and deaths averted by vaccination might be underestimated.

## Conclusions

The current meningococcal vaccination strategy adopted by the NIP in China is effective in controlling the low incidence of the disease at a low cost. The alternative vaccination strategies using non-NIP vaccines could improve the health benefits, but not in a cost-effective manner. The integration of efficient non-NIP meningococcal vaccines into NIP may be necessary for the improvement of population health, but the alternative strategies will become cost-effective upon reductions in vaccine prices. Future research should consider the dynamic model for economic evaluations of meningococcal vaccination strategies in China, and a wider population could be targeted such as adolescents and other high-risk groups. Furthermore, a well-established surveillance system across the life cycle of meningococcal cases is imperative for a more accurate assessment of the disease burden, encompassing the onset of both meningococcal meningitis and meningococcemia.

## Supplementary Material

Supplementary material_unmodified.docx

## Data Availability

The detailed data source for the model inputs are available in the article, and most parameter inputs are based on published data. The complete model structure and specific data used in the study are available upon request to the corresponding author.

## Highlights

- ***Evidencebefore this study***

Many studies examining various meningococcal vaccination strategies in different countries have been published. In China, three studies have been conducted at provincial level to evaluate the current strategy with MPV-A and MPV-AC in the NIP. All three studies concluded that the current strategy was economically favorable. However, no studies have been conducted at the national level to evaluate the alternative strategies using non-NIP vaccines, and no comparison has been performed between alternative strategies and the current strategy.

- ***Addedvalue of this study***

This study represents a pioneering effort in evaluating the cost-effectiveness of various meningococcal vaccination strategies on a national scale in China with modeling. We compared the alternative strategies using MPV-4, MCV-AC, and MCV-4 with the current strategy adopted by the NIP. We also investigated the level to which the price of the non-NIP vaccines must decrease, under current epidemiological circumstances, in order to render their inclusion in NIP cost-effective.

- ***Implicationsof all the available evidence***

In preventing meningococcal disease in China, replacing the current NIP vaccines with non-NIP vaccines could further prevent cases, and the strategy with MCV-4 could deliver the greatest health benefits. However, from a societal perspective, universal supply of non-NIP vaccines to children without charge is not cost-effective due to high prices of non-NIP vaccines. The prices of non-NIP vaccines might be decreased to facilitate inclusion in the NIP, and this would enable more cost-effective strategies.
