# Supplementary material for: Cost-effectiveness analysis of vaccination strategies against meningococcal disease for children under nine years of age in China
Source: Hum Vaccin Immunother. 2024 Feb 13;20(1):2313872. doi: 10.1080/21645515.2024.2313872 (PMC10865926; doi:10.1080/21645515.2024.2313872)
Supplement: Supplementary material_unmodified.docx [file KHVI_A_2313872_SM7314.docx]

**Supplementary material**

According to the results of the deterministic sensitivity analysis, the price of non-NIP vaccines is a crucial factor influencing ICERs. As shown in Figure S1, the ICER values increase with increasing vaccine prices. To make the corresponding alternative strategies cost-effective, the prices of MPV-4, MCV-AC, and MCV-4 vaccines should be reduced to $3.851, $9.863, and $12.568, respectively, to reach an ICER value of three times GDP per capita. When the prices of the three vaccines are reduced to $2.658, $9.350, and $12.272, respectively, the ICER value will decrease to one times GDP per capita, making the strategies very cost-effective. The reduction in vaccine prices required to decrease the ICER values from three times to one times GDP per capita is relatively small. Among the three alternative strategies, the price reduction of MCV-4 vaccine has the greatest impact on decreasing ICER values.

**Figure S1. The impact of vaccine price on the ICERs for replacements with alternative strategies**


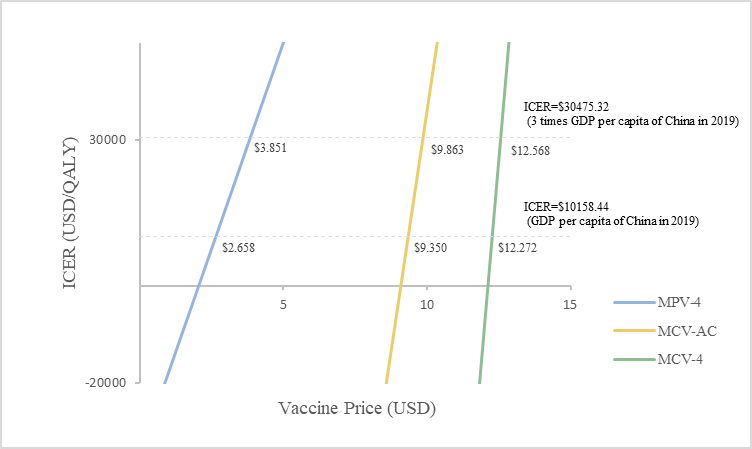


Note: The prices of MPV-A and MPV-AC remained unchanged in the *Current Practice* with which the three alternative strategies were compared. The current prices of NIP vaccines are quite low and close to the cost of production, leaving little scope for reduction in vaccine prices.^1^

Reference:

**1.** Zheng Y, Rodewald L, Yang J, et al. The landscape of vaccines in China: history, classification, supply, and price. *BMC infectious diseases.* 2018;18(1):1-8.
